# Phenanthrene impacts zebrafish cardiomyocyte excitability by inhibiting I_Kr_ and shortening action potential duration

**DOI:** 10.1085/jgp.202012733

**Published:** 2021-01-21

**Authors:** Shiva N. Kompella, Fabien Brette, Jules C. Hancox, Holly A. Shiels

**Affiliations:** 1 Division of Cardiovascular Sciences, Faculty of Biology Medicine and Health, University of Manchester, Manchester, UK; 2 Institut National de la Santé et de la Recherche Médicale, Centre de recherche Cardio-Thoracique de Bordeaux, Bordeaux, France; 3 Université de Bordeaux, Centre de Recherche Cardio-Thoracique, Bordeaux, France; 4 Institut Hospitalo-Universitaire Liryc, Electrophysiology and Heart Modeling Institute, Fondation Bordeaux Université, Pessac-Bordeaux, France; 5 School of Physiology, Pharmacology and Neuroscience, University of Bristol, Bristol, UK

## Abstract

Air pollution is an environmental hazard that is associated with cardiovascular dysfunction. Phenanthrene is a three-ringed polyaromatic hydrocarbon that is a significant component of air pollution and crude oil and has been shown to cause cardiac dysfunction in marine fishes. We investigated the cardiotoxic effects of phenanthrene in zebrafish (*Danio rerio*), an animal model relevant to human cardiac electrophysiology, using whole-cell patch-clamp of ventricular cardiomyocytes. First, we show that phenanthrene significantly shortened action potential duration without altering resting membrane potential or upstroke velocity (dV/dt). L-type Ca^2+^ current was significantly decreased by phenanthrene, consistent with the decrease in action potential duration. Phenanthrene blocked the hERG orthologue (zfERG) native current, I_Kr_, and accelerated I_Kr_ deactivation kinetics in a dose-dependent manner. Furthermore, we show that phenanthrene significantly inhibits the protective I_Kr_ current envelope, elicited by a paired ventricular AP-like command waveform protocol. Phenanthrene had no effect on other I_K_. These findings demonstrate that exposure to phenanthrene shortens action potential duration, which may reduce refractoriness and increase susceptibility to certain arrhythmia triggers, such as premature ventricular contractions. These data also reveal a previously unrecognized mechanism of polyaromatic hydrocarbon cardiotoxicity on zfERG by accelerating deactivation and decreasing I_Kr_ protective current.

## Teaser

Kompella et al. show that the petroleum-derived pollutant phenanthrene blocks the zebrafish native ERG orthologue mediating I_Kr_, thus suppressing outward protective currents initiated by premature beats and increasing the susceptibility to arrhythmia triggers.

## Introduction

The cardiac action potential (AP) is characterized by its long duration in contrast to nerve or skeletal muscle cells. Different voltage-gated ion channels shape the cardiac AP. Na^+^ channels are responsible for the rapid depolarization, the plateau phase is mediated by Ca^2+^ channels, and repolarization is due to multiple K^+^ channels. The shape depends on species and region of the heart, but the long duration is a hallmark of cardiac cells and safeguards against abnormal electrical activity, ensuring a long refractory period. Current elicited by L-type Ca^2+^ channels (I_CaL_) play critical roles in initiating the process of calcium-induced calcium release that drives cardiac excitation–contraction coupling and in providing depolarizing current that contributes to the plateau phase of cardiac APs (Bers and Despa, 2013). The rapid delayed rectifier K^+^ current (I_Kr_) is a significant contributor to cardiomyocyte AP repolarization and is underpinned by the *ether-à-go-go–related gene* (*ERG*) encoded channel (Sanguinetti et al., 1995; Trudeau et al., 1995). Due to the unique kinetics of ERG channels, they generate rapid, transient outward currents in response to premature depolarization during AP repolarization, early in diastole, therefore providing some protection from premature ventricular excitation (Lu et al., 2001; Perry et al., 2015). Pharmacological inhibition of this channel leads to prolongation of the AP, and therefore, the QT interval of the electrocardiogram. The human ERG (hERG) channel is known to be susceptible to pharmacological inhibition by diverse drugs associated with the drug-induced form of acquired long QT syndrome and associated torsades de pointes arrhythmia (Sanguinetti and Tristani-Firouzi, 2006; Vandenberg et al., 2012).

**Figure 1.**
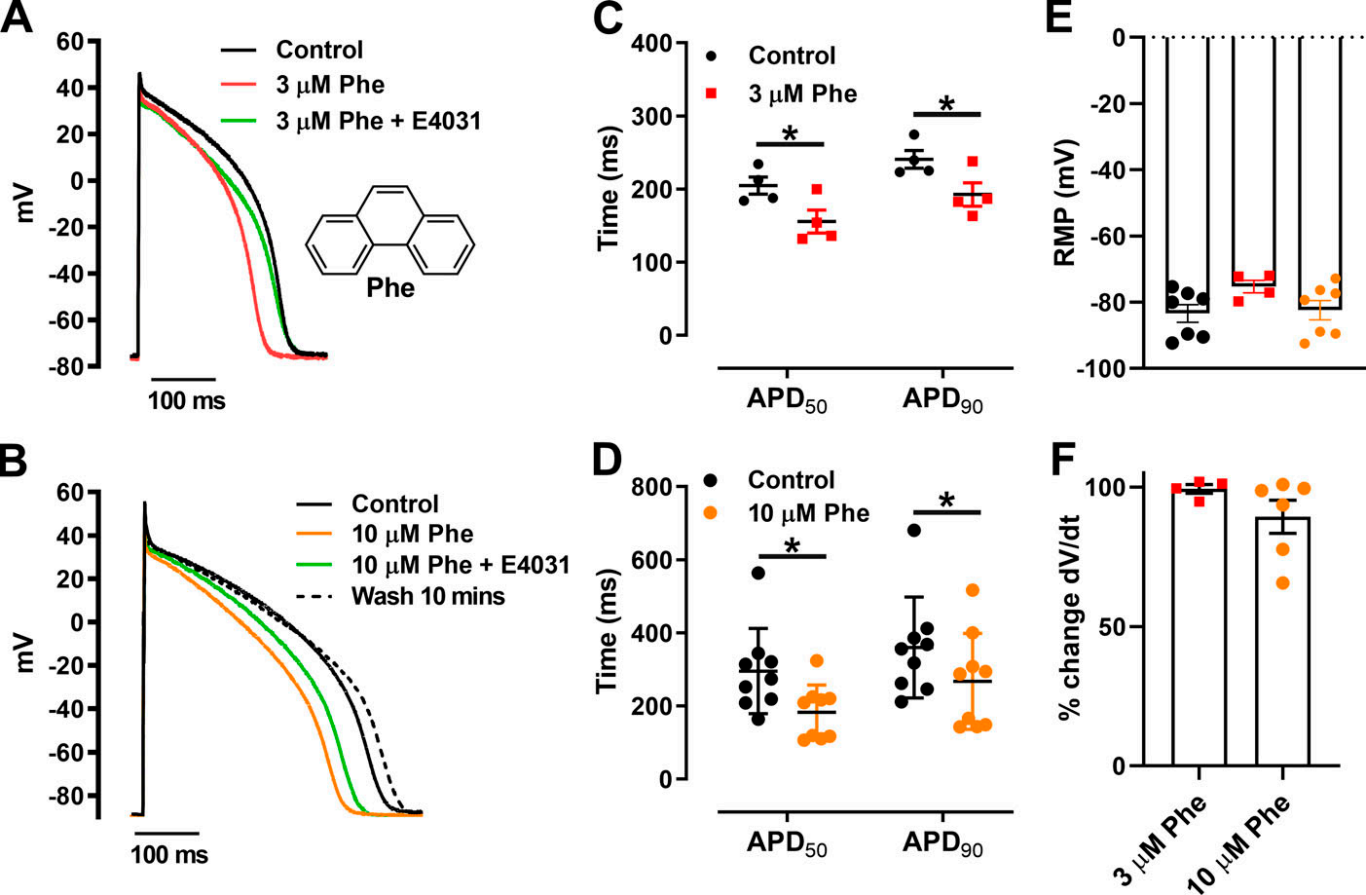
**Phenanthrene (Phe) shortens APD in zebrafish ventricular cardiomyocytes.** **(A and B)** Representative AP traces at 0.5 Hz exposed to 3 µM Phe (A) and 10 µM Phe (B; with and without 2 µM E-4031 [ERG channel blocker]) with recovery from inhibition (wash). **(A) **Inset: Molecular structure of phenanthrene. **(C and D)** Scatter plot showing mean ± SEM of APD_50_ and APD_90_ in the absence and presence of 3 µM Phe (C; *, P < 0.05; Wilcoxon test) and 10 µM Phe (D; *, P < 0.05; Wilcoxon test). **(E and F)** No significant change in resting membrane potential (RMP; E) and dV/dt was observed in presence of phenanthrene (F). All data represented as mean ± SEM (*n* = 4–9; *N* = 5).

Previous studies show that I_Kr_ and I_CaL_ are inhibited by the low-molecular-weight tricyclic polyaromatic hydrocarbon (PAH) phenanthrene (see inset in Fig. 1 A for a structural diagram of the molecule) in cardiomyocytes from marine fish species (Brette et al., 2014; Brette et al., 2017) and freshwater trout (Ainerua et al., 2020; Vehniäinen et al., 2019). In vivo PAH exposure has also been shown to reduced cardiac output in large pelagic fish (Nelson et al., 2016) and cause heart block in zebrafish embryos (Incardona et al., 2004). PAHs are present in crude oil and are derived from the burning of fossil fuels. PAHs such as phenanthrene are a ubiquitous component of both air and water pollution, but their cardiotoxicity only came to light following the catastrophic 1989 Exxon Valdez oil spill and the 2010 Deepwater Horizon blowout, which had significant toxicological effects on aquatic ecosystems (Incardona et al., 2009; Brette et al., 2014; Brette et al., 2017). In addition, there is now clear evidence of a strong correlation between air pollution and human cardiovascular diseases such as cardiac arrhythmias and stroke (Shah et al., 2013; Franklin et al., 2015; Marris et al., 2020). However, the exact mechanism(s) of detrimental effects of PAH pollutants are yet to be understood, especially in a model relevant to human cardiac electrophysiology.

The International Council for Harmonization of Technical Requirements for Pharmaceuticals for Human Use S7B cardiac safety testing guidelines specify that model species used to investigate drug effects on cardiac electrophysiology should possess ventricular APs with a high plateau phase and human-relevant repolarization mechanisms (European Medicines Agency, 2005). Mice, for example, are poorly suited to this purpose as they possess abbreviated ventricular APs and do not rely on I_Kr_ or slow delayed rectifier potassium current (I_Ks_) for repolarization (Wang et al., 1996; Nerbonne et al., 2001). By contrast, zebrafish (*Danio rerio*) exhibit a ventricular AP morphology that is similar to that in humans, with human-relevant repolarization mechanisms including I_Kr_ (Arnaout et al., 2007; Brette et al., 2008; Nemtsas et al., 2010; Abramochkin et al., 2018; Marris et al., 2020) and I_Ks_ (Abramochkin et al., 2018). The zebrafish ERG channel (zfERG) has a high level of sequence homology with hERG (Langheinrich et al., 2003; Marris et al., 2020), and zebrafish embryos have been shown to be sensitive to proarrhythmic drugs (Langheinrich et al., 2003). The zebrafish is therefore a translationally relevant model in which to study PAH cardiotoxicity.

The purpose of the present study was to characterize the effects of phenanthrene on the zebrafish ventricular AP and to study specific effects on calcium and potassium channel properties in order to determine the mechanism responsible for cardiac electrophysiological alteration in a model relevant to human cardiac physiology.

## Material and methods

All experiments were conducted in accordance with The UK Animals (Scientific Procedures) Act, 1986, and European Union Directive EU/2010/63. Local ethical approval was obtained from the University of Manchester Animal Welfare and Ethical Review Board.

### Cardiomyocyte isolation

Following stunning, the brain of the zebrafish was destroyed via pithing using a 16-gauge needle. The isolated ventricle was cannulated with a blunt 35-gauge needle through the bulbus arteriosus, tied and perfused from a height of 5 cm with a Ca^2+^-free isolation solution (see Physiological solutions) for 5 min followed by enzymatic digestion (0.65 mg/ml collagenase type 1A, 0.4 mg/ml trypsin type III, and 0.7 mg/ml BSA in 10 ml isolation solution) for 4–5 min. The ventricle was removed, cut into small pieces, and gently triturated using a Pasteur pipette to further release of single cardiac myocytes. Isolated myocytes were held at room temperature in isolation solution and used within 6 h.

### Chemicals

All solutions were prepared using ultrapure water supplied by a Milli-Q system (Millipore). Chemicals were reagent grade and purchased from Sigma-Aldrich, except for tetrodotoxin (TTX; Tocris). Purity of phenanthrene was at least 99%. Stocks of phenanthrene (25 mM) constituted in dimethyl sulfoxide (tissue culture grade; Sigma-Aldrich) were prepared fresh on the day of experiments. In all experiments, maximal DMSO concentration was <1:1,000 vol/vol.

### Whole-cell patch clamp

Electrophysiological data were recorded as previously described (Ainerua et al., 2020). Briefly, isolated myocytes were allowed to settle for ∼10 min in the recording chamber at the beginning of each trial and then perfused with control physiological solution (see Physiological solutions). Data were recorded via a Digidata 1322A A/D converter (Axon Instruments) controlled by an Axopatch 200B (Axon Instruments) amplifier running pClamp 10.3 software (Axon Instruments). Signals were filtered at 2 kHz using an eight-pole Bessel low-pass filter before digitization at 10 kHz and storage. Patch pipette resistance was typically 2–3 MΩ when filled with intracellular solution. Series resistance ranged between 5 and 10 MΩ and was compensated up to 60% for I_CaL_ voltage-clamp experiments (voltage error ≤ 2 mV).

APs were evoked using 1 ms supra-threshold current steps at a frequency of 0.5 Hz. All AP parameters were stable over the time of recording (<10 min) in controls.

**Figure 2.**
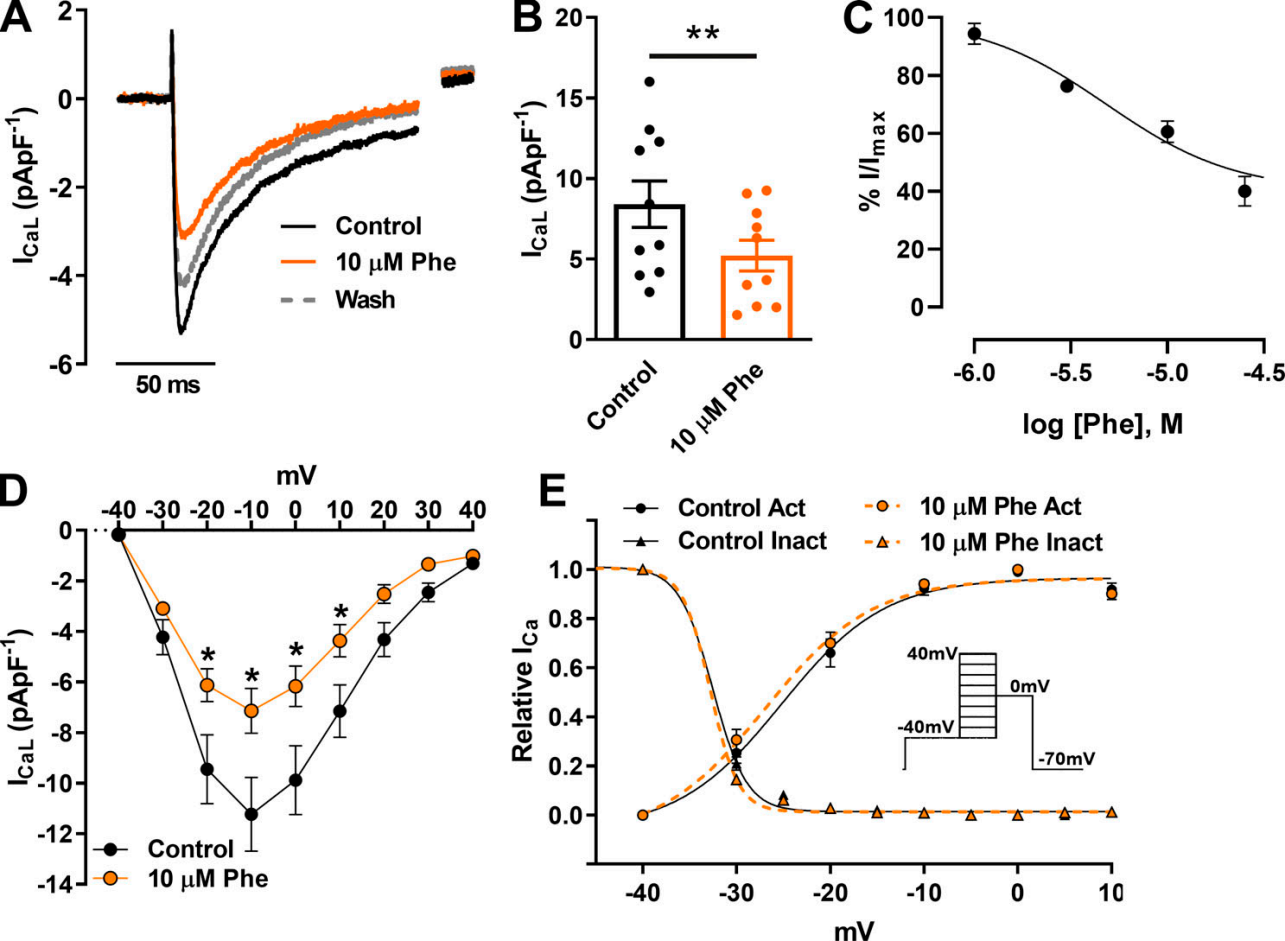
**Effect of 10 µM phenanthrene (Phe) on L-type Ca**^**2+**^** channel currents (I_CaL_) in ventricular zebrafish myocytes.** **(A)** Representative current traces. **(B)** Scatter plot showing mean ± SEM. I_CaL_ density elicited by depolarization to −10 mV (*n* = 10; *N* = 3; **, P < 0.01; Wilcoxon test) in absence (control; black) and presence of 10 µM phenanthrene (orange). Dotted line represents current trace of recovery from inhibition after 5 min washout. **(C)** Concentration–response curve of phenanthrene on I_CaL_, revealing an IC_50_ of 4.8 ± 0.8 µM (Hill slope, n_H_ = 1.3 ± 0.2). **(D)** Peak I_CaL_ density I-V plot (*n* = 6; *N* = 2; *, P < 0.05; two-way ANOVA with the Holm–Šídák post hoc analysis). **(E)** steady-state activation (G/G_max_) and inactivation (I/I_max_) obtained using (inset) double-pulse protocol in the absence (control; black) and presence of 10 µM phenanthrene (orange; *n* = 6; *N* = 2). Act, activation; Inact, inactivation.

I_CaL_ was elicited using a double-pulse protocol (see Fig. 2 C, inset) that consisted of 2 s duration prepulses from −40 to +40 mV in 10-mV steps, a 20-ms inter-pulse interval at −40 mV, and a test pulse to 0 mV for 2 s. A prepulse to −40 mV from holding potential of −70 mV (500 ms) was used to inactivate Na^+^ and T-type Ca^2+^ current before the double-pulse protocol. I-V and steady-state activation curves were obtained from currents elicited during prepulse voltage steps. Availability (steady-state inactivation) curves were obtained from currents elicited during test pulse. The stimulus frequency was 0.1 Hz.

I_Kr_ was activated by a test pulse to +20 mV (2.5 s) to fully activate K^+^ channels (as determined in preliminary experiments) and measured as the tail current at −40 mV (4.5 s) at a stimulation frequency of 0.1 Hz. A prepulse to −40 mV from a holding potential of −70 mV (500 ms) was used to inactivate any Na^+^ and T-type Ca^2+^ current. The instantaneous I-V relation was measured from currents recorded using an AP-like voltage-clamp protocol at a frequency of 0.5 Hz with an initial depolarizing step from −70 mV to +30 mV for 500 ms followed by repolarizing ramp (from +30 mV to −70 mV) of 150 ms. I_Kr_ rapid transient currents were generated using a paired AP-like voltage-clamp protocol as described above with depolarizing stimuli to 0 mV at increasing intervals (7.5 ms). I_Kr_ transient currents were obtained following each condition (control, phenanthrene, and E-4031) within the same cell. Peak transient currents obtained in presence of 2 µM E-4031 were subtracted from corresponding currents in both control and phenanthrene exposure. Peak I_Kr_ transients for each cell under control conditions were then normalized to the maximal current transient. Peak I_Kr_ transients obtained in presence of 3 µM phenanthrene are represented as percentage response of the corresponding control transient.

Other I_K_ were activated by a depolarizing pulse to +40 mV (4 s) to fully activate K^+^ channels (as determined in preliminary experiments) at a frequency of 0.1 Hz, following I_Kr_ inhibition (see below). A prepulse to −40 mV from holding potential of −70 mV (500 ms) was used to inactivate any Na^+^ and T-type Ca^2+^ current (Abramochkin et al., 2018).

### Physiological solutions

The isolation solution contained (in mM) 100 NaCl, 10 KCl, 1.2 KH_2_PO_4_, 4 MgSO_4_, 50 taurine, 20 glucose, and 10 HEPES, with pH set to 6.9 with KOH. The composition of the extracellular physiological solution (Ringer’s) used for electrophysiology contained (in mM) 150 NaCl, 3.5 KCl, 1.5 MgCl_2_, 2 CaCl_2_, 10 glucose, and 10 HEPES, with pH set to 7.7 with NaOH. To avoid overlapping ion currents during voltage-clamp recording of I_CaL_, I_Kr_, and other I_K_, this solution was modified. For I_CaL_ measurement, KCl was replaced by CsCl to inhibit K^+^ channels, and TTX (0.5 µM) was used to inhibit Na^+^. For barium current (I_BaL_) measurement, CaCl_2_ was replaced by equimolar BaCl_2_ in the above solution. For I_Kr_ recording, TTX (0.5 µM), nifedipine (10 µM), and glibenclamide (10 µM) were included in the Ringer’s solution to inhibit Na^+^, Ca^2+^, and ATP-sensitive K^+^ channels, respectively. The concentration of TTX used in this study to inhibit I_Na_ current, albeit low, is in accordance with previous studies (Haverinen et al., 2007) that showed greater sensitivity of TTX to Na^+^ channels in fish cardiomyocytes (in the nanomolar range) compared with mammalian cardiomyocytes (in the micromolar range). For recording net I_K_, 2 µM E-4031 was included in the external solution to inhibit the zfERG channel. The remaining K^+^ currents represent all outward I_K_ except I_Kr_. Solutions were locally superfused over the cell using a 6–1 manifold and VC-6 six-channel valve controller perfusion system (Warner Instruments) to switch between various solutions.

Pipette solutions were optimized for each electrophysiological recording condition. For AP measurement, the pipette solution contained (in mM) 10 NaCl, 140 KCl, 5 MgATP, 0.025 EGTA, 1 MgCl_2_, and 10 HEPES, with pH adjusted to 7.2 with KOH. For I_Ca_ measurement, the pipette solution contained (in mM) 130 CsCl, 15 TEA-Cl, 5 MgATP, 1 MgCl_2_, 5 Na_2_-phosphocreatine, 5 EGTA, 10 HEPES, and 0.03 Na_2_GTP, with pH adjusted to 7.2 with CsOH. CsCl and TEA-Cl were included to inhibit K^+^ currents. For I_Kr_ and other I_K_ measurements, EGTA concentration was increased to 5 mM in the AP pipette solution.

### Data analysis

AP amplitude was measured as the difference between the overshoot and the resting membrane potential. The maximum rate of rise of the AP (change in voltage [dV] as a function of the maximum change in time [dt_max_], in V/s) was calculated by differentiation of the AP upstroke using Clampfit software. AP duration (APD) was measured as the duration from the overshoot to three different percentages of repolarization (30%: APD_30_; 50%, APD_50_; and 90%, APD_90_). Currents are expressed as current density by normalizing to cell size (pApF^−1^). I_CaL_ amplitude was measured as the difference between peak and the end of depolarizing pulse current. For steady-state activation curve, conductance (G) of I_CaL_ for each test potential was determined using

$$G\;=\;I_{o}/(V_{m}-V_{rev}),$$

where I_o_ is the peak amplitude of I_CaL_ at each prepulse potential (V_m_), and V_rev_ is the apparent reversal potential obtained by fitting the ascending limb of I-V plot through the zero-current axis.

The activation parameter at each potential was determined as

$$G/G_{max},\;$$

where G_max_ was the largest observed value of G during the protocol. The inactivation parameter at each potential was determined as I/I_max_, where I is the peak amplitude of I_CaL_ at each test potential and I_max_ was the largest observed value of I during the protocol.

Steady-state activation (G/G_max_) and inactivation (I/I_max_) curves from each cell were fitted with the Boltzmann equation,

$$G/G_{max}\;=1/[1+e^{\left[(V_{0.5}-V_{m})/k\;\right]}]$$

$$I/I_{max}\;=1-1/[1+e^{\left[(V_{0.5}-V_{m})/k\;\right]}\mathrm{],}$$

where V_0.5_ is the potential at which half-maximal activation or inactivation of I_CaL_ was obtained, V_m_ is the membrane potential, and k is the slope factor for the relationship. Individual V_0.5_ and k values obtained from above Boltzmann fit were averaged and presented as mean ± SEM in Table 1.

**Table 1.** Steady-state activation and inactivation parameters of I_CaL_ in the absence and presence of 10 µM phenanthrene (Phe)

Activation			Inactivation	
	Control	10 µM Phe	Control	10 µM Phe
V_0.5_	−23.6 ± 1.3 mV	−24.9 ± 1.1 mV	−31.8 ± 0.1 mV	−32.0 ± 0.1 mV
k	5.1 ± 0.2	5.1 ± 0.2	1.4 ± 0.2	1.1 ± 0.1

Parameters were obtained by fitting with Boltzman equation of individual activation (G/G_max_) and inactivation (I/I_max_) curves as described in Materials and methods. V_0.5_, half-maximal voltage of activation and inactivation; k, slope factor. All values are presented as means ± SEM (*n* = 6; *N* = 2).

The deactivation rate of I_Kr_ was quantified by fitting tail currents with the following bi-exponential equation below using Clampfit 10.3 software:

$$I=A_{f}e^{\left(-t/\tau_{f}\right)}+A_{s}e^{\left(-t/\tau_{s}\right)}+C.$$

*I* represents the current amplitude at time *t*; *A_f_* and *A_s_*, respectively, represent the amplitude of fast and slow deactivating current components, fitted with time constants τ_fast_ (*τ_f_*) and τ_slow_ (*τ_s_*); C represents any residual unfitted current.

The fraction of fast-deactivating current was obtained using the equation

$$A_{f}/(A_{f}+A_{s}),$$

where *A_f_* and *A_s_* are the amplitudes of the fitted component of fast-deactivating and slow-deactivating current, respectively.

The concentration–response relation was fitted using unweighted nonlinear regression, using the following equation:

$$E=1/\left\{1+10^{\left[\left(LogIC_{50}-X\right)\right]}{}^{*n_{H}}\right\}.$$

E is the response, X is the phenanthrene concentration, *n*_H_ is the slope factor (Hill coefficient), and IC_50_ is the antagonist concentration giving 50% inhibition of the maximal response.

### Statistical analysis

Data are reported as mean ± SEM. Due to a nonnormal distribution, paired data were analyzed with a Wilcoxon signed-rank test (percentage data) or a Mann–Whitney test with P values as indicated within the text and figure legends using GraphPad Prism 8 software. A two-way ANOVA with Holm–Šídák post hoc analysis was used for I_CaL_ I-V plot. Samples sizes for animals are given as *N* and for myocytes as *n* in the text and figure legends.

### Online supplemental material

**Figure S1.**
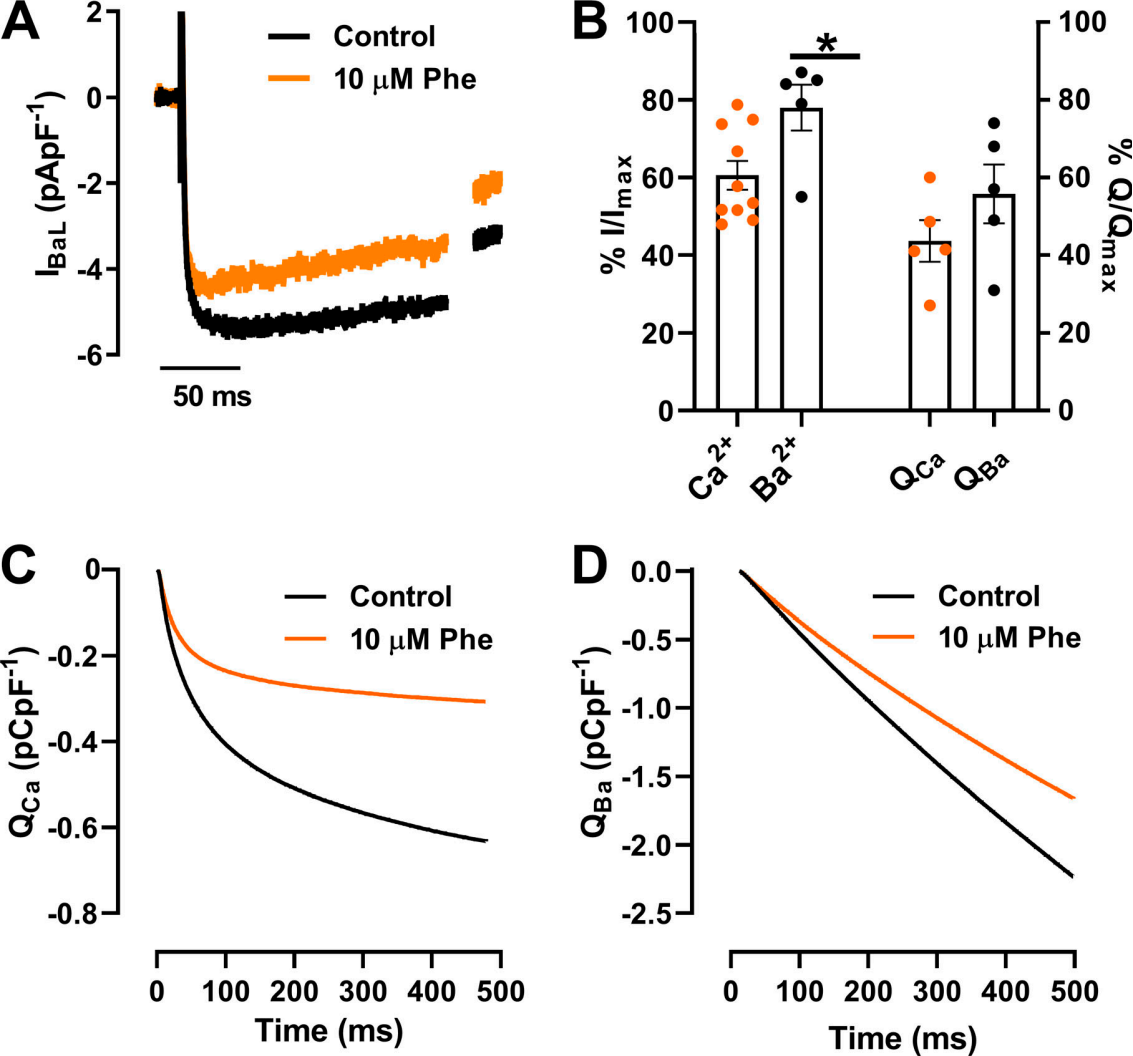
**Effect of phenanthrene (Phe) on Ba**^**2+**^** currents (I_BaL_) of L-type Ca**^**2+**^** channels.** **(A)** Representative current traces of I_BaL_ elicited by depolarization to −10 mV in the absence (control; black) and presence of 10 µM phenanthrene (orange). **(B)** Bar graph showing mean ± SEM percentage change in peak currents (I) and total charge (Q) of Ca^2+^ (*n* = 5–10; *N* = 2) and Ba^2+^ (*n* = 5; *N* = 3) in the absence and presence of 10 µM phenanthrene (*, P < 0.05; Mann–Whitney Test). **(C and D)** Representative traces of cumulative charge transfer as integral of time for calcium currents (C) and barium currents (D) in the absence and presence of 10 µM phenanthrene.

**Figure S2.**
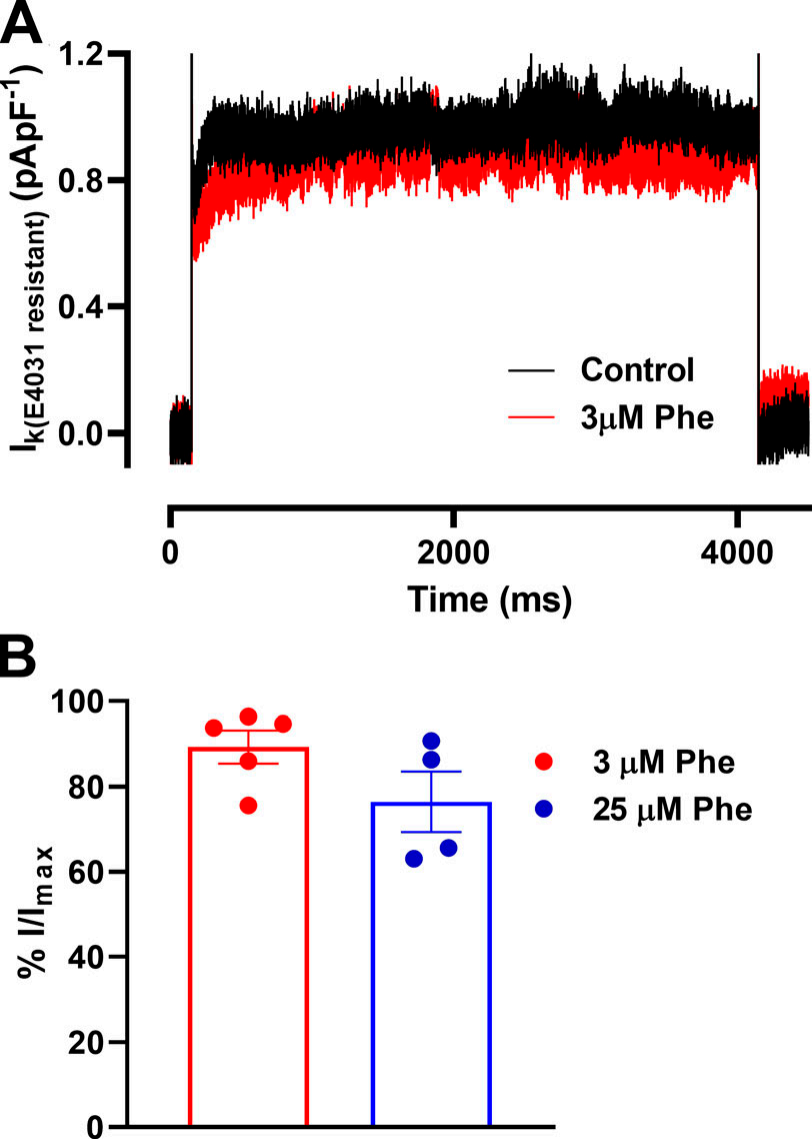
**Effect of phenanthrene (Phe) on other potassium current (I_K_).** **(A)** Representative current traces of net I_K_ elicited by depolarization to +40 mV in the absence (control; black) and presence of 3 µM phenanthrene (red). External solution contained 2 µM E-4031 to block I_Kr_. **(B)** Bar graph showing mean ± SEM percentage change in peak currents in the absence and presence of 3 µM (*n* = 5; *N* = 2) and 25 µM (*n* = 4; *N* = 2) phenanthrene.

Fig. S1 shows the effect of phenanthrene on the barium current (I_BaL_) through L-type Ca^2+^ channels. Fig. S2 shows the effect of phenanthrene on the potassium current (I_K_) resistant to E4031.

## Results

### Phenanthrene shortens APD in zebrafish ventricular cardiomyocytes

We recorded APs using a current clamp in the whole-cell configuration. Fig. 1 A shows shortening of the APD in response to 3 µM phenanthrene. Phenanthrene application at both 3 and 10 µM had no significant effect on resting membrane potential (Fig. 1 E) and upstroke velocity (dV/dt_max_; Fig. 1 F), triangulation (measured as difference in APD at 30% and 90% of repolarization), or beat-to-beat variability rate from control values (data not shown). In contrast, APD_50_ and APD_90_ (measured at 50% and 90% of repolarization, respectively) were significantly decreased by acute superfusion of 3 µM phenanthrene (Fig. 1 A). This is in contrast to previous studies in bluefin tuna and brown trout in which AP prolongation was observed (Brette et al., 2017; Ainerua et al., 2020). Subsequent application of 3 µM phenanthrene with E-4031, a selective I_Kr_ inhibitor (at a supramaximal concentration of 2 µM), led to prolongation of APD_90_ (Fig. 1 A, green line). AP shortening was greater at 10 than 3 µM phenanthrene, leading to significant reduction of both APD_50_ and APD_90_ (Fig. 1 D). Application of E-4031 in the presence of 10 µM phenanthrene led to prolongation of APD_90_ (Fig. 1 B), suggesting that I_Kr_ was not fully blocked by phenanthrene at concentrations between 1 and 10 µM. Mean data indicate a significant reduction in APD_50_ and APD_90_ in 3 µM (*n* = 4; *N* = 2) and 10 µM (*n* = 9; *N* = 5) phenanthrene, respectively (Fig. 1, C and D; P < 0.05; Wilcoxon test). Partial recovery in both APD_50_ and APD_90_ was observed following 10 min washout (Fig. 1 B, dotted line). Taken together, these data suggest that I_K1_ (inwardly rectifying K^+^ current), the current responsible for resting membrane potential, and I_Na_, the current responsible for the upstroke of AP, are not modified by phenanthrene, and that the likely ionic currents affected by phenanthrene are I_CaL_ and K^+^ currents, resulting in an overall decrease in APD.

### Phenanthrene inhibits L-type Ca^2+^ current

Fig. 2 A shows that phenanthrene significantly decreased I_CaL_ in zebrafish ventricular myocytes, as described previously in other fish species (Brette et al., 2017). Partial recovery from inhibition was observed following 5 min wash (Fig. 2 A). Mean data at −10 mV showed a 39 ± 4% inhibition (Fig. 2 B; *n* = 10; *N* = 3; P < 0.05; Wilcoxon test). Construction of a concentration–response relation yielded an IC_50_ of 4.5 ± 0.8 µM (Fig. 2 C; n_H_ = 1.3 ± 0.2; *n* = 5–10; *N* = 3). To examine this effect across a range of voltages, we used a double-pulse protocol (Fig. 2 E, inset), which showed significant voltage-dependent inhibition of I_CaL_ currents within the range of −20 to +10 mV in presence of 10 µM phenanthrene (Fig. 2 D; *n* = 6; *N* = 2; P < 0.05; two-way ANOVA with Holm–Šídák post hoc test). Peak I_CaL_ was elicited at −10 mV, and this showed no shift in the presence of phenanthrene. This is further confirmed by the lack of significant differences in steady-state activation and inactivation parameters, including window current, between control and phenanthrene conditions (Fig. 2 E). To better understand the mechanism of phenanthrene inhibition of I_CaL_, we repeated our studies with Ba^2+^ as the charge carrier (Fig. S1). Phenanthrene had less of an inhibitory effect on peak Ba^2+^ currents compared with peak Ca^2+^ currents (only 22 ± 6%, compared with 39 ± 4%; P < 0.05; Mann–Whitney test; Fig. S1 B; *n* = 5; *N* = 3). There was a trend for a reduction in the total charge (Q) transferred by Ba^2+^ compared with Ca^2+^ (Fig. S1, B–D). These data indicate a Ca^2+^-dependent inhibition by phenanthrene. However, further experiments are required to determine the exact mechanism. Together these results indicate that APD shortening in response to phenanthrene exposure may be accounted for by reduced I_Ca_ density. However, the AP plateau phase involves a delicate balance between the activity of L-type Ca^2+^ channels and K^+^ channels. Previous studies of phenanthrene on ventricular cardiomyocytes from several fish species showed significant AP prolongation linked to inhibition of I_Kr_ (Brette et al., 2014; Brette et al., 2017; Ainerua et al., 2020). Thus, we next assessed the effect of phenanthrene upon I_Kr_.

### Phenanthrene inhibits I_Kr_ but not other I_K_ currents in zebrafish cardiomyocytes

**Figure 3.**
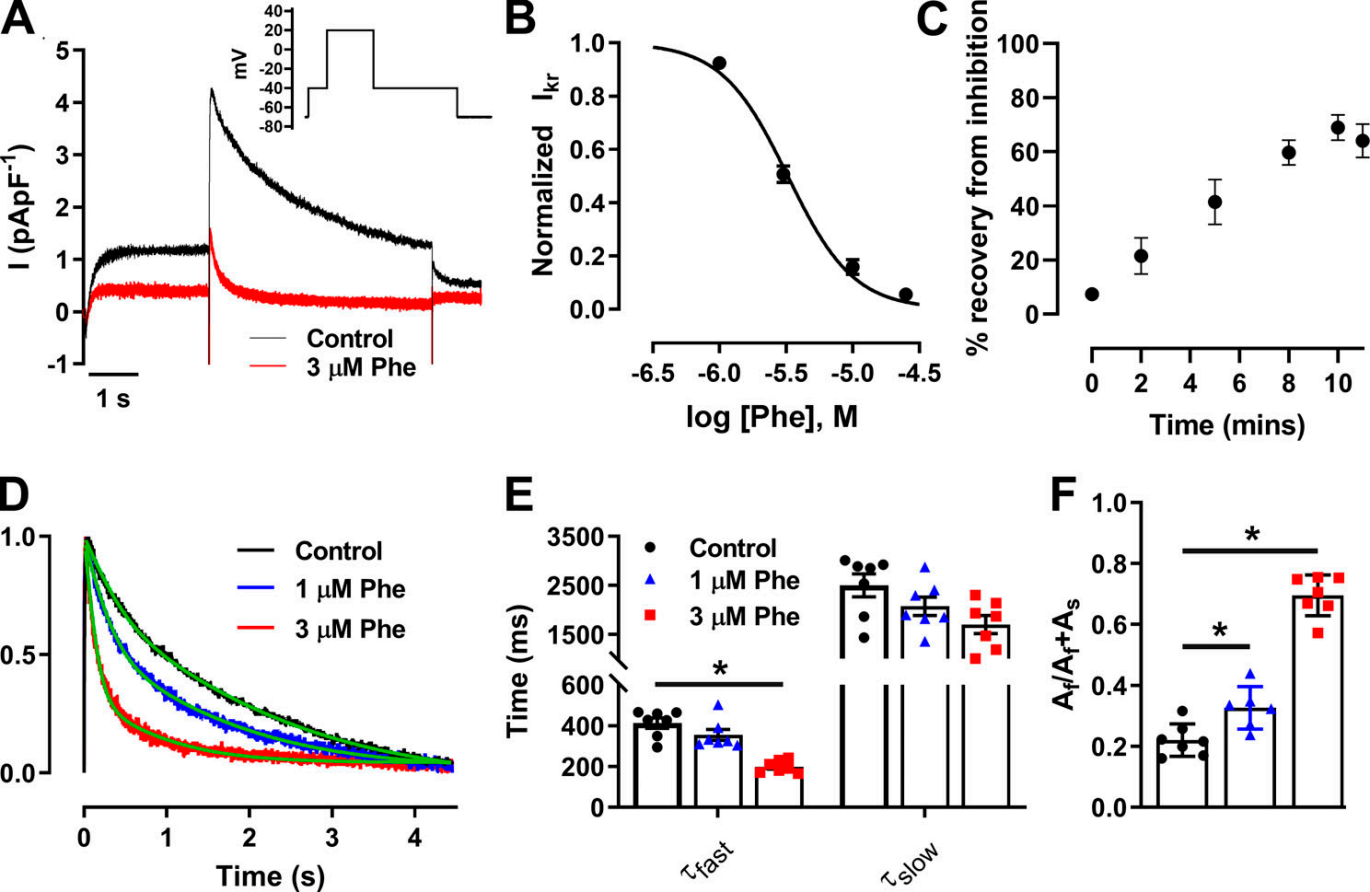
**Phenanthrene (Phe) inhibits the native zfERG channel (I_Kr_) and increases rate of channel deactivation.** **(A)** Representative I_Kr_ peak tail current trace recorded in absence (black) and presence of 3 µM phenanthrene (red; inset) elicited by voltage protocol. **(B)** Concentration–response curve of phenanthrene on peak I_Kr_ tails, revealing an IC_50_ of 3.3 ± 0.2 µM (Hill slope, n_H_ = 1.8 ± 0.2). **(C)** Time-course plot showing a maximum of 69 ± 5% recovery from inhibition by 10 µM phenanthrene after 10 min of washout (*n* = 6; *N* = 2). **(D)** Representative I_Kr_ tails (normalized to peak) in the absence (black) and presence of 1 µM (blue) and 3 µM (red) phenanthrene, fitted to a standard double exponential curve (green), to evaluate rate of current decay. **(E and F)** Bar plot presenting time-constant values of fast- and slow-deactivating current components (τ_fast_ and τ_slow_, respectively; E; P < 0.01) and proportion of amplitude of fast-deactivating current component (A_f_) as a fraction of total amplitude of fast- and slow-deactivating current component (A_f_ + A_s_) obtained from fitting (F) show significant decrease in τ_fast_ and corresponding increase in A_f_ component with increasing concentration in phenanthrene (*n* = 7; *N* = 2; *, P < 0.05; Wilcoxon test).

Zebrafish ventricular cardiomyocytes exhibited robust peak I_Kr_ tail currents (Fig. 3 A) at −40 mV following a voltage protocol incorporating activating commands to +20 mV (Fig. 3 A, inset), as previously described (Nemtsas et al., 2010), with a mean current density of 2.4 ± 0.3 pApF^−1^ (*n* = 9; *N* = 2). Phenanthrene at 3 µM, 10 µM, and 25 µM induced a significant 49 ± 3% (*n* = 9; *N* = 3), 84 ± 3% (*n* = 6; *N* = 3), and 94 ± 1% (*n* = 5; *N* = 2; P < 0.05; Wilcoxon test) decrease of peak I_Kr_ tail current, respectively. I_Kr_ tail amplitude was reduced in a concentration-dependent manner in response to phenanthrene exposure with an IC_50_ of 3.3 ± 0.2 µM and a Hill coefficient of 1.8 ± 0.2 (Fig. 3 B; *n* = 5–9; *N* = 3). A plot of the time-course curve showed a maximum of 69 ± 5% recovery from inhibition by 10 µM phenanthrene after 10 min of washout (Fig. 3 C; *n* = 6; *N* = 3). Phenanthrene also increased rate of decay of peak I_Kr_ tail currents in a concentration-dependent manner (Fig. 3 D). A significant reduction in time constant of fast-deactivating current component, τ_fast_ (Fig. 3 E; *n* = 8; *N* = 2) was associated with an increase in the contribution of fast-deactivating current component A_f_ (Fig. 3 F; *n* = 8; *N* = 2).

To investigate whether other I_K_ were modified by phenanthrene, we recorded remaining I_k_ during E-4031 superfusion before and after phenanthrene application. No significant effects at 3 and 25 µM were observed, strongly suggesting that I_Kr_ is the only I_K_ modified by phenanthrene (*n* = 4–5; *N* = 2; Fig. S2, A and B).

**Figure 4.**
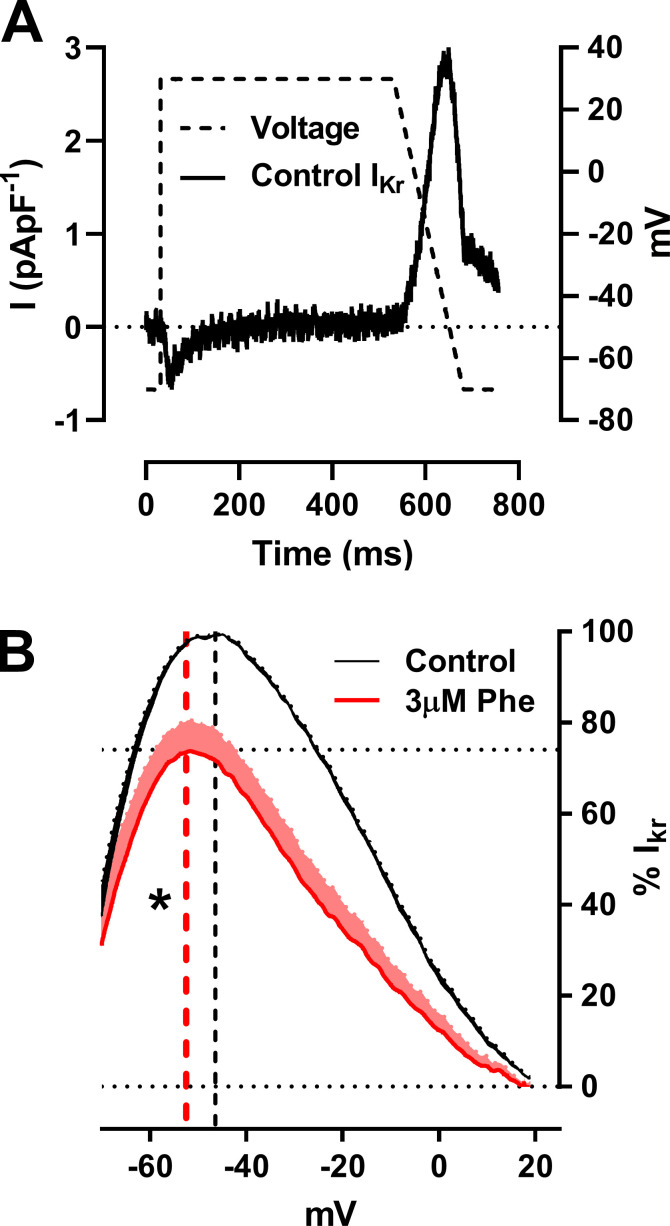
**Phenanthrene (Phe) significantly shifts the voltage of repolarizing I_Kr_ during AP-like command by −6 mV.** **(A)** Representative traces of ventricular AP-like command waveform protocol used to elicit shown I_Kr_ current trace. **(B)** Mean ± SEM percentage response of I_Kr_ exhibiting an inhibition of 24 ± 7% in the presence of 3 µM phenanthrene (red). Mean peak voltage during the repolarization phase is shown in the corresponding colored dashed line exhibiting a significant shift of ∼6 mV from −46.4 mV for control to −52.5 mV in the presence of 3 µM phenanthrene (*n* = 6; *N* = 2; *, P < 0.05; Wilcoxon test).

To further investigate the effects of phenanthrene on I_Kr_ under more physiologically relevant conditions, we used an AP-like voltage clamp protocol (Fig. 4 A). I_Kr_ current showed a voltage dependence similar to that of the fully activated I-V relation (Fig. 4 B) due to rapid recovery from inactivation as AP repolarization proceeds. Therefore, zebrafish I_Kr_ during the AP-like protocol is very similar to that recorded from mammalian ventricular myocytes (Hancox et al., 1998; Zaza et al., 1998). The instantaneous I-V relation during the repolarizing ramp (from +30 to −70 mV) was bell-shaped with peak current at −46.4 ± 1.2 mV (*n* = 7; *N* = 2; Fig. 4 B). Interestingly, 3 µM phenanthrene had less (P < 0.05; Mann–Whitney test) of an inhibitory effect on the instantaneous peak current (24 ± 7% of peak current; Fig. 4 B) when compared with I_Kr_ peak-tail currents (49 ± 3% of peak current). In addition, phenanthrene induced a significant shift of −6 mV in mean peak I-V (from −46.4 ± 1.2 to −52.5 ± 1.5 mV; Fig. 4 B; P < 0.05; Wilcoxon test; *n* = 7; *N* = 2). This result raised the possibility that I_Kr_ may be less protective during the late repolarization phase following exposure to phenanthrene. To investigate this further, we tested the effect of phenanthrene upon rapid I_Kr_ transient outward currents elicited by a paired ventricular AP-like command waveform protocol.

### Phenanthrene inhibits protective I_Kr_ transient currents

**Figure 5.**
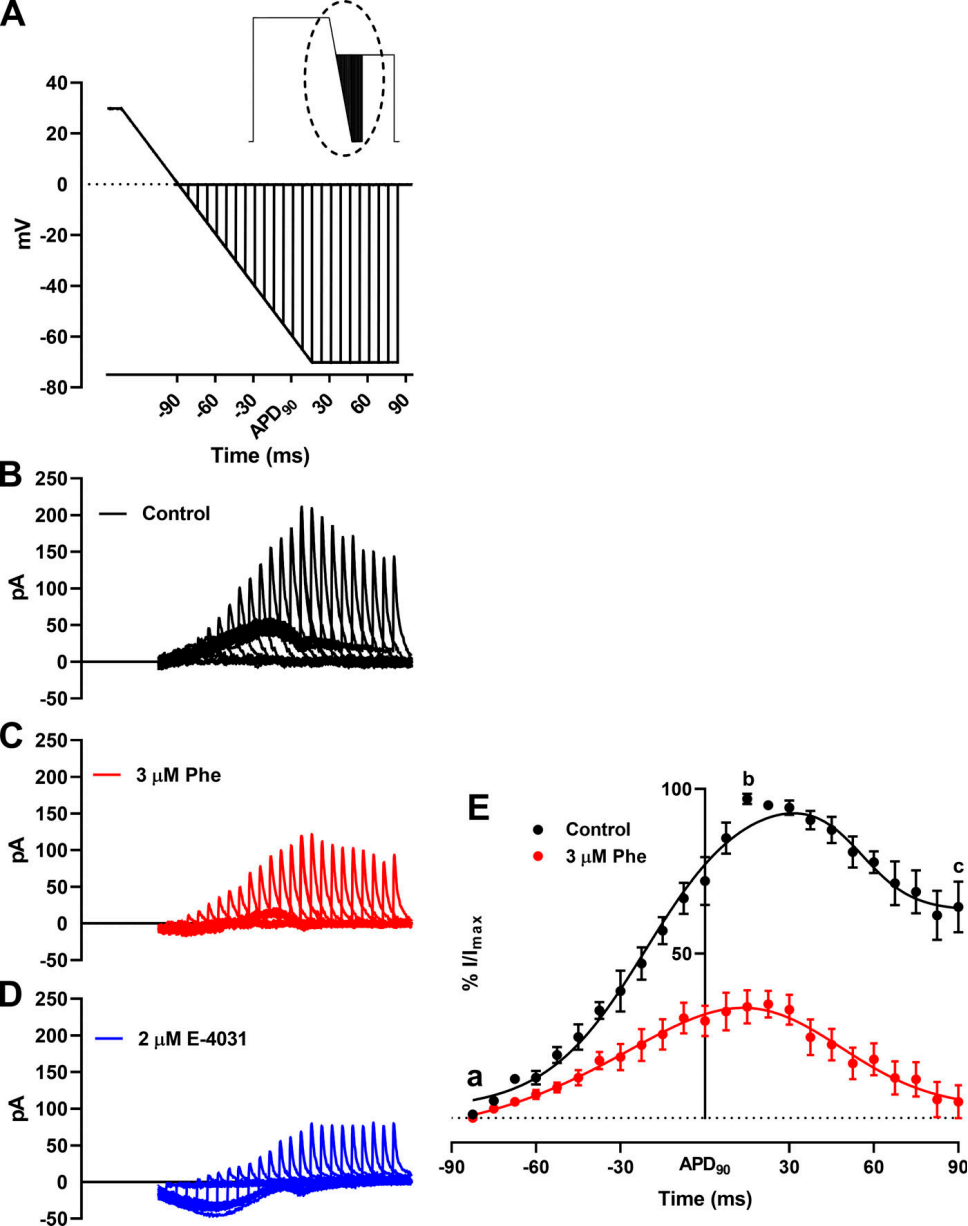
**Effect of phenanthrene (Phe) on response of I_Kr_ to premature stimulation.** **(A)** Magnified view of the dotted area of (inset) paired ventricular AP-like command waveform protocol. **(B–D)** Representative trace of families of transient currents elicited corresponding to each depolarization in the absence (B; black) and presence (C) of 3 µM phenanthrene (red) and 2 µM E-4031 (D; blue). **(E)** Percentage response of peak I_Kr_ transients in the absence (black) and presence of 3 µM phenanthrene (red). Peak I_Kr_ transients for each cell under control conditions were normalized to the maximal current transient during the protocol. Peak I_Kr_ transients in the presence of 3 µM phenanthrene (red) are represented as percentage response relative to its corresponding percentage control transient. First, peak, and last transient obtained are labeled a, b, and c, respectively. All data represented as mean ± SEM (*n* = 7 or 8; *N* = 3).

Fig. 5 A, inset, shows the double pulse protocol used to record I_Kr_ in response to premature depolarizing stimuli, introduced from APD_90_ −90 ms to APD_90_ +90 ms (Fig. 5 A). Under control conditions, I_Kr_ transient current peaked at 15 ms after APD_90_ (Fig. 5 E, b). This current is considered protective against cardiac arrhythmia (Perry et al., 2015), and 3 µM phenanthrene induced significant inhibition of this peak transient current over the entire range of depolarizing stimuli (Fig. 5 E). Notably, the magnitude of the peak I_Kr_ transient current inhibition (63 ± 6%; Fig. 5 E, b), is similar to that of I_Kr_ tail currents. Together, these results indicate that protective I_Kr_ transient currents were blunted by phenanthrene.

## Discussion

Pollutants disrupt the fine-tuned electrical activity of the heart and increase the risk of cardiac arrhythmias and sudden cardiac death (Shah et al., 2013; Franklin et al., 2015). In this study, we show that phenanthrene, a low-molecular-weight tricyclic PAH, decreases APD in zebrafish. We show that phenanthrene decreases L-type Ca^2+^ current and the zfERG-mediated I_Kr_ current while accelerating I_Kr_ deactivation. Lastly, we show that the protective role of I_Kr_ (in the form of rapid I_Kr_ transients) is diminished by this pervasive air and water pollutant.

### Disruption of ionic current induces AP shortening by pollutant phenanthrene

Air pollution affects human cardiovascular function and can lead to death (Marris et al., 2020). Cardiac arrhythmias represent a significant proportion of these deaths (European Medicines Agency, 2017). Phenanthrene is present in the aquatic environment primarily due to crude oil spills and is also present in the atmosphere as a volatile substance and in association with particulate matter (e.g., diameter of 2.5 µm or less; Tsapakis and Stephanou, 2005; Incardona et al., 2014; Marris et al., 2020). The devastating oceanic oil spills focused studies on the impact of PAHs on fish cardiovascular function, whereas comparable studies on mammals associated with air pollution have lagged behind (Marris et al., 2020). In freshwater brown trout and various pelagic fishes, phenanthrene significantly prolonged the AP at both the cellular and whole heart levels (Brette et al., 2017; Ainerua et al., 2020). The prolongation of AP in these studies is attributed to inhibition of the I_Kr_ current. This differs from the present study in zebrafish showing a significant AP shortening (Fig. 1). A recent study found no effect of phenanthrene on the APD in rainbow trout ventricular cardiomyocytes at concentrations between 0.3 and 30 µM, but did find significant APD shortening after acute exposure to an alkylated phenanthrene (retene) at concentrations between 1 and 10 µM (Vehniäinen et al., 2019).

The relative APD shortening or lengthening in response to an agent depends largely on its inhibitory potency on inward (mainly Na^+^ and Ca^2+^) and outward (mainly K^+^) ionic conductances (Hancox et al., 2008). Thus, it is interesting to note that in both the rainbow trout study (Vehniäinen et al., 2019) and our present study, phenanthrene exerted significant inhibitory activity on I_Kr_ amplitude (Fig. 3, A–C), which is predicted to prolong APD (Brette et al., 2017; Ainerua et al., 2020). Our finding of APD shortening instead of prolongation despite the significant impact on I_Kr_ implicates significant inhibition of I_CaL_ in response to phenanthrene exposure. Phenanthrene has been reported to inhibit L-type Ca^2+^ channel current and reduce the intracellular Ca^2+^ transient, thus affecting the contractility of ventricular myocytes in a number of marine fishes and in the freshwater brown trout (Brette et al., 2017; Ainerua et al., 2020). However, pharmacological potencies varied greatly in these studies, with ∼30% inhibition exhibited by 5 µM phenanthrene in bluefin tuna (Brette et al., 2017), whereas similar inhibition was observed only at 30 µM phenanthrene in brown trout (Ainerua et al., 2020). We show that phenanthrene exhibited a maximum inhibition of 60 ± 5% of the zebrafish L-type calcium current at 25 µM concentration (Fig. 2 C) with an IC_50_ value of 4.5 µM (Fig. 2 C), which may account for AP shortening following phenanthrene exposure. It is important to note that fish are more reliant on transsarcolemmal Ca^2+^ influx during excitation–contraction coupling than mammals (Shiels and Galli, 2014), which impacts the density of inward and outward currents during an AP across species. This is consistent with preliminary data reported in Marris et al. (2020) showing AP prolongation in sheep ventricular cardiomyocytes in the presence of phenanthrene. Thus, the AP shortening observed in our study could highlight important species-specific differences in the balance of Ca^2+^ influx to K^+^ efflux during the AP and the differential pharmacological potencies of phenanthrene toward Ca^2+^ and K^+^ channels. Such differences need to be kept in mind when extrapolating findings from zebrafish to humans.

### Effects of phenanthrene on I_Kr_ and other I_K_

Inhibition of I_Kr_ by various molecules has previously been shown to induce 2:1 atrio-ventricular block and arrhythmias in zebrafish (Langheinrich et al., 2003; Milan et al., 2003; Incardona et al., 2004; Mittelstadt et al., 2008). Atrio-ventricular block and bradycardia have also been reported following phenanthrene exposure in in vivo studies using zebrafish embryos (Incardona et al., 2004; Incardona et al., 2005; Zhang et al., 2013). Interestingly, in the present study, phenanthrene exhibited differential inhibition of the potassium currents. Notably, only 24 ± 7% inhibition in the presence of 3 µM phenanthrene (Fig. 4 B) was observed during the AP-like ramp protocol (Fig. 4 A), compared with greater (50%) inhibition observed for peak-tail currents (Fig. 3 B). A significant (−6 mV) shift in peak repolarization current voltage (Fig. 4 B) during the AP-like ramp protocol was also observed. Contrary to this, the antimalarial drug halofantrine (which shares structural similarity with phenanthrene) exhibited similar inhibition of hERG channel under both AP-clamp and tail-current voltage protocol (Mbai et al., 2002). Halofantrine is a potent (21.6 nM IC_50_) hERG channel inhibitor in mammals that has been proposed to require channel gating to occur (Mbai et al., 2002). Under ventricular AP-clamp, halofantrine’s inhibitory action was greatest during phases 2 and 3 of the AP (Mbai et al., 2002). The lower potency (3.3 µM IC_50_) of I_Kr_ inhibition observed here is consistent with the smaller size of phenanthrene than halofantrine, as this would be expected to result in fewer simultaneous binding contacts with residues on the ERG/I_Kr_ channel. Alanine mutagenesis has revealed key interactions between halofantrine and aromatic (Y652, F656) residues of the hERG canonical drug binding site (Sánchez-Chapula et al., 2004).

In parallel with this study on zebrafish, structure–functional experiments on phenanthrene interactions with the human zfERG counterpart, hERG, have been undertaken to determine the nature of the phenanthrene interaction site(s) on the channel. These have demonstrated voltage and time dependence of block and a dependence of inhibition on intact inactivation gating (Al-Moubarak et al., 2020; Al-Moubarak et al. 2019. Proc Physiol Soc 43, PC016). These features are compatible with the actions of a range of known gating-dependent, pore-blocking inhibitors (Sanguinetti and Mitcheson, 2005; Hancox et al., 2008). Interestingly, phenanthrene inhibition of hERG appears to be sensitive to mutation of an S5 aromatic residue (Al-Moubarak et al., 2020; Al-Moubarak et al. 2019. Proc Physiol Soc 43, PC016).

Recently, I_Ks_ has been recorded in isolated zebrafish cardiomyocytes, although it differs from that in ventricular myocytes from mammalian species, with faster activation, most likely due to a reduced contribution of KCNE1 (Abramochkin et al., 2018). Similar to I_Kr_, inhibition or loss-of-function mutations of I_Ks_ are associated with long QT phenotypes in mammals (Veerman et al., 2013). Indeed, I_Ks_ is thought to provide “repolarization reserve” and is thus cardio-protective against arrhythmias but via a mechanism that is distinct from the protection from premature stimulation observed here for I_Kr_ (Varró and Baczkó, 2011). The lack of effect of phenanthrene on zebrafish E4031 resistant–I_k_ reported here indicates that this mechanism remains intact during exposure (Fig. S2), which may contribute to the absence of APD prolongation observed in this study. Moreover, this finding supports the notion that the APD shortening effect of phenanthrene was attributable to decreased calcium currents without concomitant activation of outward potassium currents.

### (Patho)physiological significance

The lack of AP prolongation or increased beat-to-beat variability seen here with phenanthrene is not consistent with increased proarrhythmic risk linked to repolarization delay. To our knowledge, there are at present no published studies on the impact of phenanthrene on ventricular APs and underlying ionic conductances in a human experimental system. Thus, the translational relevance of these observations to the human heart remains to be established. Regardless, the suppression of the response of I_Kr_ to premature stimulation (Fig. 5 E) is consistent with increased vulnerability to premature electrical excitation. Phenanthrene significantly increased the rate of deactivation of I_Kr_ in a concentration-dependent manner (Fig. 3, D–F), and this acceleration of deactivation together with the channel-blocking effect of the drug may account for the reduced ability of I_Kr_ to generate rapid transient currents compared with control (Fig. 5 E). Under normal conditions, this transient current would be expected to counter premature depolarization (Lu et al., 2001; Perry et al., 2015), and such a response would be expected to be impaired in the presence of phenanthrene. Zebrafish APD shortening combined with increased I_Kr_ deactivation could lead to abbreviation of post-repolarization refractoriness, increasing susceptibility to arrhythmias. Post-repolarization refractoriness acts as a protective mechanism in the heart by preventing multiple compound APs from occurring (i.e., it limits the frequency of depolarization and therefore excitation rate; Grunnet et al., 2008). Consistent with this notion, accelerated I_Kr_/hERG deactivation (as a response to extracellular acidosis) has previously been shown to reduce the protective role of I_Kr_ early in diastole and decrease AP excitation threshold in a human ventricular AP model (Du et al., 2010). This is also pharmacologically exemplified by a recent study wherein excessive AP abbreviation by type 2 hERG activator, ICA-105574, was found to be proarrhythmic (Perry et al., 2020). Phenanthrene might be anticipated to produce a similar effect, which could be further augmented under conditions of reduced I_Kr_ contribution to repolarization and protective transient currents, as would occur with loss-of-function *hERG* mutations (long QT syndrome 2; Perry et al., 2016). Further evidence of a key role for I_Kr_ deactivation in influencing arrhythmia susceptibility comes from a recent study with the small molecule RPR260243 that only slows hERG channel deactivation kinetics (Shi et al., 2020). This compound exhibited anti-arrhythmic properties by increasing post-repolarization refractoriness and attenuated the irregular actional potential firing by dofetilide (I_Kr_ inhibitor). Future experiments are now warranted to determine the overall effect of phenanthrene on arrhythmia susceptibility at the intact zebrafish heart level, using an in vivo or ex vivo heart model.

Plasma and urine concentrations of PAHs in humans are in the nanomolar range, with a recent study reporting phenanthrene concentrations of ∼200 nM in human blood (Huang et al., 2016). However, it is important to note that phenanthrene, like most tricyclic PAHs, is highly lipophilic (logP [octanol/water] coefficient of ∼4.4), explaining the higher tissue than plasma concentrations observed in exposure studies (Carls et al., 1999; Heintz et al., 1999; West et al., 2014). The low micromolar potency of phenanthrene on both I_Kr_ (3.3 µM; Fig. 3 C) and I_CaL_ (4.5 µM; Fig. 2 C) reported here is therefore concerning, as concentrations as high as 3 µM have been reported in animal tissues (Camacho et al., 2012; Dhananjayan and Muralidharan, 2013).

### Conclusion

The present study demonstrates the cardiotoxic effect of the air and water pollutant phenanthrene in an animal model with human cardiac relevance. This work demonstrates inhibition of zebrafish L-type Ca^2+^ channel current (IC_50_ = 4.5 µM) and I_Kr_ (IC_50_ = 3.3 µM) and also identifies acceleration of I_Kr_ deactivation as a significant action of phenanthrene, accompanied by a reduction in I_Kr_ protective transient currents at a concentration (3 µM) previously reported in human tissues (Jacob and Seidel, 2002). Interestingly, a comprehensive study looking at hERG inhibition of ∼4,800 tricyclic aromatic compounds revealed an average IC_50_ value of ∼2 µM (Ritchie et al., 2011). Therefore, phenanthrene and other PAHs in petroleum-based pollution could have detrimental effects on human cardiac health, warranting future studies to evaluate the impact of phenanthrene exposure on large animal models.
